# A Modified Synthetic Pathway for the Synthesis of so far Inaccessible N1-Functionalized Tetrazole Ligands – Synthesis and Characterization of the 1D Chain-Type Spin Crossover Compound [Fe(3ditz)_3_](BF_4_)_2_

**DOI:** 10.1002/ejic.201201062

**Published:** 2013-01-17

**Authors:** Danny Müller, Christian Knoll, Berthold Stöger, Werner Artner, Michael Reissner, Peter Weinberger

**Affiliations:** [a] Institute of Applied Synthetic Chemistry, Vienna University of TechnologyGetreidemarkt 9/163-AC, 1060 Vienna, Austria, Fax: +43-1-58801-16299; [b] X-ray Center, Vienna University of TechnologyGetreidemarkt 9, 1060 Vienna, Austria; [c] Institute of Chemical Technologies and Analytics, Vienna University of TechnologyGetreidemarkt 9/164-SC, 1060 Vienna, Austria; [d] Institute of Solid State Physics, Vienna University of TechnologyWiedner Hauptstrasse 8–10/138, 1040 Vienna, Austria

**Keywords:** Ligand design, N ligands, Spin crossover, Magnetic properties, Iron

## Abstract

A modified phase-transfer-catalyst-assisted synthetic pathway was developed that widens the pool of accessible 1-substituted tetrazoles, which are possible ligands for iron(II) spin-crossover compounds. Within the family of α,ω-bis(tetrazol-1-yl)alkanes, a series of ligands and their respective iron(II) spin-crossover compounds were synthesized and structurally and spectroscopically characterized in the past. The classical route to prepare these ligands is based on the respective amino-precursors. Hence the pool of accessible compounds is limited by the commercial or synthetical availability of α,ω-diaminoalkanes. Furthermore, the concomitant transformation to the tetrazole moieties turns out to be easier for diamino-alkanes with an even number of carbon atoms than for those with an odd number. In line with this observation, the shortest odd-numbered homologues such as 1,1-bis(tetrazol-1-yl)methane (*1ditz*) and 1,3-bis(tetrazol-1-yl)propane (*3ditz*) were inaccessible so far. In this paper, we report the successful preparation and characterisation of the classically inaccessible 1,3-bis(tetrazol-1-yl)propane (*3ditz*) and of its spin-crossover complex [Fe(*3ditz*)_3_](BF_4_)_2_, which features an abrupt and almost complete spin transition at *T*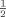 = 159 K. The single-crystal X-ray structure of the low-spin and the high-spin species is presented. The magnetic data are supported by variable-temperature IR, UV/Vis/NIR, and ^57^Fe Mössbauer spectra.

## Introduction

In the quest for technologically applicable iron(II) spin-crossover (SCO) compounds, a rational design of the coordination complexes with appropriate magneto-optical properties^1^ is of utmost importance. It is a matter of fact that several factors (i.e., ligand type, solvent used, synthesis conditions, etc.) govern the key properties of spin-crossover complexes, such as the thermal spin transition (*T*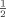) and the abruptness of the spin switching between the high-spin (HS) and the low-spin (LS) species.^2^ Therefore, intense research effort is focused on the elucidation of a structure–property relationship. Despite of the existence of rare predictive models, for example, for 1D chain-type triazole-based Fe^II^ coordination polymers,^3^ it is impossible to predict the spin-transition behavior of almost any new SCO compound, because the empirical trends established thus far are typically specific for a single class of compounds only.^4^ To shed light on the impact of the structural details of any chosen ligand on the imposed SCO properties, we prepared a homologue series of ligands to derive trends within their SCO complexes.^5^ The synthetic strategy usually applied was derived from the classical tetrazole synthesis established by Franke et al.^6^ This reaction scheme allows for the synthesis of aryl- as well as alkyl-substituted mono-N1- or di-N1,N1′-tetrazoles. Thus, a whole library of ligands could be synthesized and was the basis for a series of mononuclear and polynuclear iron(II) SCO compounds.^5^,^7^ Especially, the class of bridging α,ω-bis(tetrazol-1-yl)alkanes (*nditz; n is the number of carbon atoms in the alkyl spacer*) was of great interest, because the variation of the spacer length between the two coordinating tetrazole moieties created a fascinating playground for the investigation of the impact of the used solvent and the counteranion on the SCO behavior of the complexes.^8^ The iron(II) complexes of 1,2-bis(tetrazol-1-yl)ethane (*2ditz*) and the 1,2-bis(tetrazol-1-yl)propane (*btzp*) feature a chain-type coordination-polymer structure with a rather weak cooperativity of the spin-switching iron(II) coordination centers,^7^ whereas the coordination of Fe(II) with 1,4-bis(tetrazol-1-yl)butane (*4ditz*) gives rise to threefold interpenetrated 3D networks, which show a two-step abrupt spin-transition behavior. The latter compound was studied in depth to show the impact of the size of the counteranion as well as of the solvent, and this study elucidated the drastic effects these modifications have.^8^ The homologues with longer spacers (*5ditz*–*9ditz*) revealed an unusual parity effect, which is due to the number and parity of carbon atoms in the alkyl spacer. Because the flexibility of these ligands increases with the spacer length, the magnetic properties of their respective iron(II) complexes are not promising with respect to applicable compounds.5c As the cooperativity of the spin switching of the iron(II) coordination centers is a crucial prerequisite for abrupt spin transitions, the preparation of the shorter α,ω-bis(tetrazol-1-yl)alkanes, that is, the so far inaccessible 1,3-bis(tetrazol-1-yl)propane (*3ditz*), was a synthetic challenge in recent time. As this “missing link” in the series of bridging α,ω-bis(tetrazol-1-yl)alkanes (*nditz*) was interesting with respect to systematic variations of the ligand that shed light on its influences on the spin-transition properties, we put effort into developing alternative synthetic strategies to prepare this compound. As this alternative protocol allows preparing the target molecules from bromoprecursors rather than from aminoprecursors, the shortest bridging ligand (*1ditz*) may become accessible as well, but this will be the subject of further investigations that go beyond the scope of this paper.

## Results and Discussion

As the classical synthetic pathway by Franke et al.,^6^ which starts from the corresponding 1,3-diaminopropane, never yielded the desired product, a new synthetic approach that starts from the 1,3-dibromopropane and makes use of a phase-transfer catalyst was developed. In principle, the use of phase-transfer catalysts for the synthesis of tetrazoles has been known for decades. The list of tetrazole compounds prepared by this method ranges from industrial-application compounds such as 5-(benzylmercapto)-1*H*-tetrazole (synthesized with hexadecyltrimethylammonium bromide as phase-transfer catalyst)^9^, to several biologically active tetrazole compounds of the family of angiotensin-II inhibitors (i.e., irbesartan, cardesartan, losartan, and valsartan), which are C5-substituted,^10^ to a variety of C5,N1-disubstituted tetrazoles.^11^ However, on the basis of the above mentioned synthetic protocols, there is no established synthetic pathway for the efficient synthesis of N1-substituted tetrazoles such as the N1-substituted 1,3-bis(tetrazol-1-yl)propane (*3ditz*). So far, only the N2-substituted and the mixed N1,N2-substituted 1,3-bis(tetrazol-1-yl)propane were reported in the literature.^12^ For synthetic details see the Experimental Section.

### Molecular and Crystal Structure of *3ditz*

**Figure 1:**
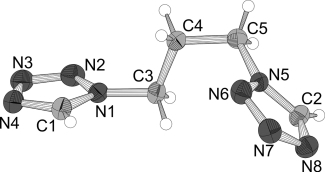
Ellipsoid plot of the molecular structure of *3ditz*. C and N atoms are represented by light and dark grey ellipsoids, respectively, drawn at 75 % probability levels. H atoms are represented by white spheres of arbitrary radius.

*3ditz* crystallizes in the space group *P*2_1_/*n*, and one crystallographically unique molecule is located on a general position. The two tetrazole units are in *anti* (C1, N1–N4) and in *gauche* conformation (C2, N5–N8) to the propyl group, respectively (Figure 1).

The *3ditz* molecules are connected through nonclassic C–H**···**N hydrogen bonds, which involve the H atoms of the tetrazole units (Table 1).

**Table 1:** Nonclassic C–H···N hydrogen bonds in the crystal structure of *3ditz*, which involve hydrogen atoms of the tetrazole rings

Atoms	C–H [Å]	H···N [Å]	C–N [Å]	C–H···N [°]
C1–H1**···**N8[a]	0.949(10)	2.385(10)	3.3016(10)	162.1(9)
C2–H2**···**N2[b]	0.968(10)	2.477(10)	3.3935(10)	157.7(9)

[a] –*x*, 1 – *y*, –*z*.

[b] 3/2 –*x*, *y* + 1/2, 1/2 –*z*.

**Figure 2:**
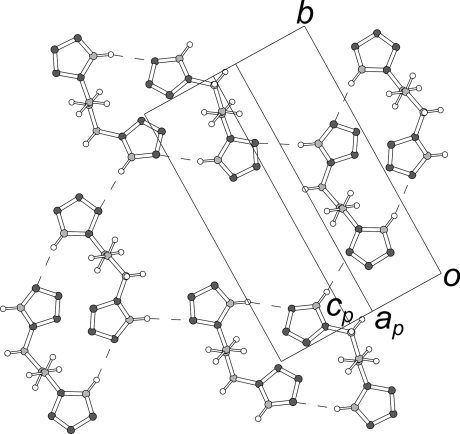
Layers of hydrogen-bonded 3*ditz* molecules parallel to (10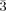) projected on the layer plane.

Pairs of *3ditz* molecules that are related by inversions are connected by two C1–H1**···**N8 bonds. These pairs are connected by C2–H2**···**N2 bonds to infinite 2D sheets extending parallel to (10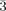) (Figure 2).

**Figure 3:**
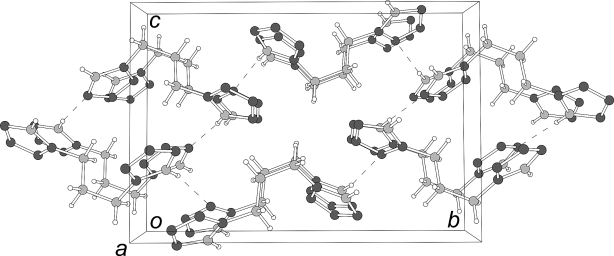
Packing diagram of the crystal structure of *3ditz*.

The sheets are stacked along [10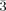] and connected by van der Waals interactions and possibly by weak hydrogen bonds involving aliphatic hydrogen atoms. A packing diagram of the crystal structure is given in Figure 3.

### Synthesis of the Complex [Fe(*3ditz*)_3_](BF_4_)_2_

A straightforward complexation reaction yielded the new spin-crossover coordination polymer [Fe(*3ditz*)_3_](BF_4_)_2_, which exhibits an almost complete and abrupt spin transition at *T*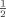 = 159 K (see the Exp. Section for synthetic details). The obtained powder product was used for all physicochemical characterizations, whereas single crystals were used for the X-ray crystal structure determination. Calculated powder diffraction patterns derived from the experimental single-crystal X-ray diffraction data were compared with measured XRPD (X-ray powder diffraction) data to prove that both, the crystals as well as the powder product, were the same compound. For details, see Figure S1 in the Supporting Information.

### Crystal Structure of [Fe(*3ditz*)_3_](BF_4_)_2_

**Figure 4:**
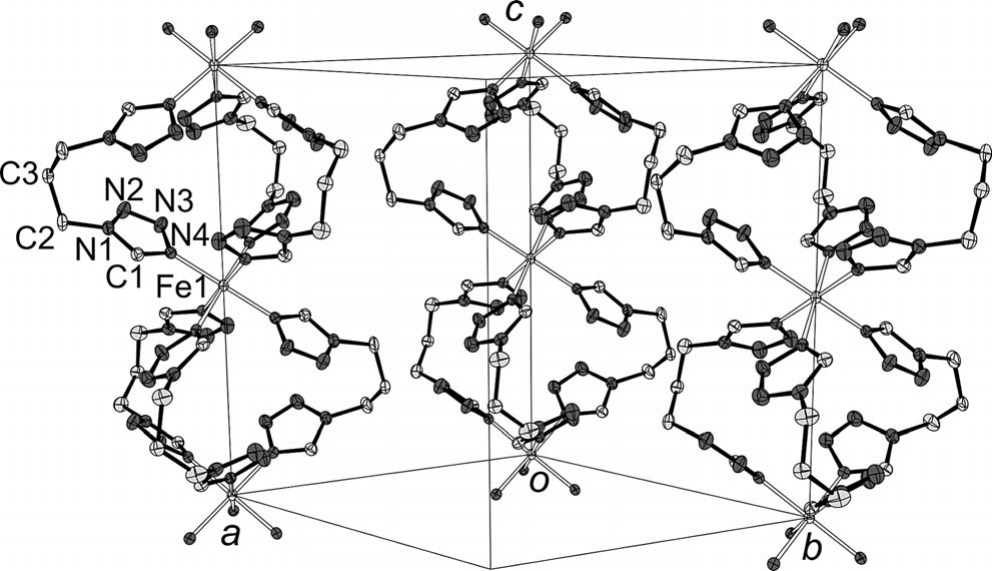
Perspective projection of [Fe(*3ditz*)_3_](BF_4_)_2_ showing the 1D chain structure. Color codes as in Figure 1, Fe^2+^ ions are white. Ellipsoids are drawn 50 % probability levels. H atoms and the disorder of the ligands are omitted for clarity.

Despite the spin transition at 159 K, the structures of [Fe(*3ditz*)_3_](BF_4_)_2_ at 100 and at 200 K are crystallographically equivalent. The complex crystallizes in the space group *P*
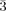
*c*1, and the structure features one crystallographically unique Fe^2+^ ion located on a site with a 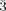 symmetry and one unique *3ditz* ligand. The Fe^2+^ centers are coordinated by six *3ditz* molecules acting as bridging ligands. Thus infinite [Fe(*3ditz*)_3_]^2+^ chains are formed, which extend along [001] with a *p*
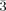
1*c* symmetry (Figure 4).

The coordination sphere of the Fe^2+^ ion is made up of six equivalent N4 atoms located at the corners of a practically regular octahedron (symmetry 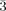). The Fe–N bond lengths at 100 [6 × 1.9947(14) Å] and 200 K [6 × 2.180(2) Å] are in good agreement with Fe^2+^ in its low-spin and high-spin state, respectively.

**Figure 5:**
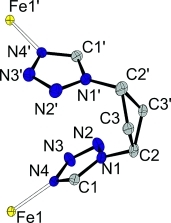
Disordered *3ditz* ligand connecting two Fe^2+^ ions. Color code: C is represented by grey, N by blue and Fe by yellow ellipsoids. Primed and nonprimed atoms are related by a twofold axis. C3 and C3′ are half-occupied. H atoms are omitted for clarity.

The aliphatic spacers of the *3ditz* ligands are disordered around twofold axes parallel to <100>. The central methylene (–CH_2_–) group had to be refined as a split position, whereas the disorder of the outer methylene groups is reflected by enlarged atomic displacement parameters (ADP) (Figure 5).

**Figure 6:**
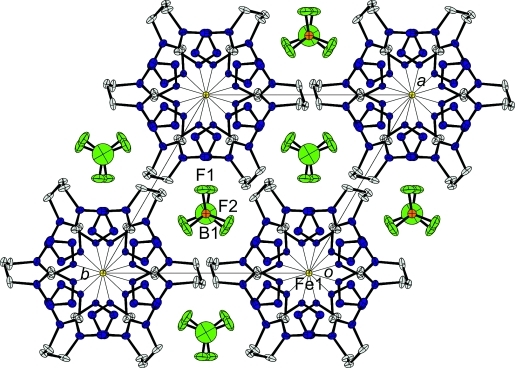
View of the crystal structure of [Fe(*3ditz*)_3_](BF_4_)_2_ along [001]. Color code as in Figure 5; B is represented by red and F by green ellipsoids. H atoms and the disorder of the ligands and BF_4_^–^ anions are omitted for clarity.

The space between the [Fe(*3ditz*)_3_]^2+^ chains is filled by BF_4_^–^ anions located on a site with symmetry 3 (Figure 6).

The BF_4_^–^ anions are disordered: Two different orientations are related by a pseudo mirror symmetry with a plane parallel to (001). The occupation ratio of both orientations was determined as 0.742:0.258(5).

[Fe(*3ditz*)_3_](BF_4_)_2_ can be considered isostructural^13^ to the positional isomer [Fe(*btzp*)_3_](ClO_4_)_2_7a and, irrespective of disorder, likewise to the two-carbon homologue [Fe(*2ditz*)_3_](BF_4_)_2_.7b As in the title complex, the ligands in [Fe(*btzp*)_3_](ClO_4_)_2_ are disordered around the twofold axes parallel to <100>. In contrast, the ligands in [Fe(*2ditz*)_3_](BF_4_)_2_ are disordered by pseudo-symmetry, namely, by mirroring at planes parallel to {100}. As a consequence, the disorder affects only the aliphatic spacer in [Fe(*3ditz*)_3_](BF_4_)_2_ and [Fe(*btzp*)_3_](ClO_4_)_2_, but it affects the whole ligand in [Fe(*2ditz*)_3_](BF_4_)_2_. On the other hand, the disorder of the BF_4_^–^ and ClO_4_^–^ anions is equivalent in all three structures. In contrast to [Fe(*2ditz*)_3_](BF_4_)_2_, we did not observe any streaking or broadening of reflections in the diffraction pattern of [Fe(*3ditz*)_3_](BF_4_)_2_, which would point to correlated disorder or small domains. However, it has to be noted that the crystal under investigation was tiny, and diffuse scattering may have been unobserved because of a lack of intensity.

Surprisingly, because of a more corrugated conformation of the *3ditz* ligand in comparison to the shorter ligands *2ditz* and *btzp*, the Fe–Fe distances along the cationic chains are significantly shorter in the *3ditz* complex [7.1325(11) Å at 100 K for *3ditz* compared to 7.293(4) and 7.273(1) Å for *2ditz* and *btzp*, respectively], which translates to a shorter lattice parameter *c* = 2*d*(Fe–Fe). The additional space needed for the extra methylene group in *3ditz* is provided by a less dense packing of the cationic chains compared to the *2ditz* complex, which also results in an increase of the lattice parameters *a = b* [10.9153(4) Å for *3ditz* compared to 10.178(2) Å for *2ditz*]. The packing is even less dense in [Fe(*btzp*)_3_](ClO_4_)_2_ [*a = b* = 11.030(2) Å], which is due to the space needed for the methyl group of the ligand and for the larger anions. In total and as expected, the unit cell volume increases with the size of the ligands and of the anions: [Fe(*2ditz*)_3_](BF_4_)_2_ (1308.6 Å^3^), [Fe(*3ditz*)_3_](BF_4_)_2_ [1471.88(14) Å^3^], [Fe(*btzp*)_3_](ClO_4_)_2_ [1532.6(4) Å^3^]. For the crystal structure data of both the free *3ditz* ligand and the corresponding Fe^2+^ complex see Table 2.

**Table 2:** Crystal and diffraction data for *3ditz* and [Fe(*3ditz*)_3_](BF_4_)_2_

	*3ditz*	[Fe(*3ditz*)_3_](BF_4_)_2_ at 100 K	[Fe(*3ditz*)_3_](BF_4_)_2_ at 200 K
Empirical formula	C_5_H_8_N_8_	FeC_15_H_24_N_24_B_2_F_8_	FeC_15_H_24_N_24_B_2_F_8_
Formula weight	180.2	770.0	770.0
Crystal system	monoclinic	trigonal	trigonal
Space group	*P*2_1_/*n*	*P* 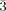 c1	*P* 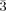 c1
*a* /Å	5.1113(2)	10.9153(4)	11.0529(3)
*b* /Å	15.0899(6)	10.9153(4)	11.0529(3)
*c* /Å	10.3860(4)	14.2650(11)	14.5434(5)
*β* /°	92.628(2)	120	120
*V* /Å^3^	800.22(5)	1471.88(14)	1538.68(8)
*T* /K	100	100	200
*Z*	4	2	2
*μ* /mm^–1^	0.110	0.621	0.594
Reflections collected	13026	32967	40179
Independent reflections	2929	1178	1192
*R*[*I* > 3σ*I*], *wR*[all], GooF	0.0350 (2464 refl.), 0.0462, 2.42	0.0353 (828 refl.), 0.0366, 1.77	0.0502 (888 refl.), 0.0753, 3.56

### Magnetic and Spectroscopic Characterisation of [Fe(*3ditz*)_3_](BF_4_)_2_

**Figure 7:**
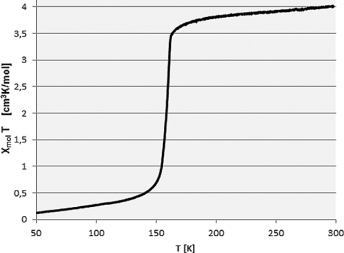
Molar magnetic susceptibility of [Fe(*3ditz*)_3_](BF_4_)_2_ between 50 K and 300 K at an external field strength of *H* = 1 T.

The magnetic susceptibility of [Fe(*3ditz*)_3_](BF_4_)_2_ was measured between 50 K and 300 K, which revealed an almost complete and abrupt spin-transition behavior at *T*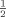 = 159 K (see Figure 7).

The astonishing abruptness of the spin transition is due to a strong cooperative effect among the spin-switching iron(II) coordination centers, and it can be explained by the significantly shorter Fe^II^–Fe^II^ distance alongside the 1D chain-type coordination polymer in comparison to the homologous compound [Fe(*2ditz*)_3_](BF_4_)_2_ (i.e., 7.13 Å compared to 7.29 Å, respectively, at 100 K).7a Furthermore, the three helically twisted bridging *3ditz* ligands form a stiffer packing motif than the shorter *2ditz* ligands in the corresponding [Fe(*2ditz*)_3_](BF_4_)_2_ compound, thus preventing the kind of shock-absorber effect of the bridging ligands that impairs the cooperativity of the iron(II) centers in the [Fe(*2ditz*)_3_](BF_4_)_2_ compound.^14^ Therefore, the title complex is another example for spin-crossover compounds with per se flexible ligands that yield an abrupt spin transition-behavior, which is mainly due to packing effects rather than to the stiffness of the ligand itself.

**Figure 8:**
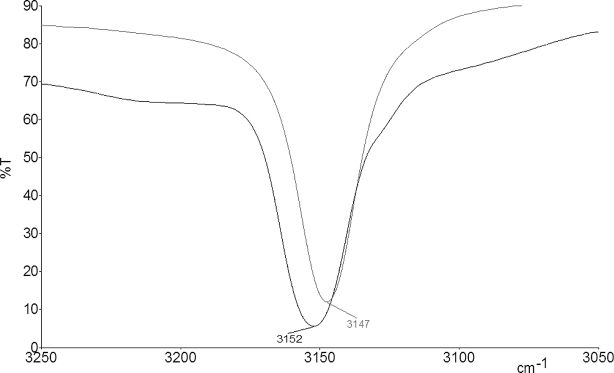
Variable-temperature MIR spectra: a comparison of the ν_CH_ band of [Fe(*3ditz*)_3_](BF_4_)_2_ at 93 K (black) and at 298 K (grey).

The results of the magnetic measurements are supported by independent spectroscopic measurements, such as variable-temperature far-range IR (FIR), mid-range IR (MIR), and UV/Vis/NIR spectroscopy. As the spin transition between high-spin and low-spin iron(II) has a drastic impact on the bond strength of the Fe–N4 bond, it also has an impact on the strengths of the neighboring N4–C1 bonds and even on the next-neighboring C1–H1 bonds. Especially, the bond-strength change of the tetrazole C–H group upon spin transition of the iron(II) can be detected by variable-temperature MIR spectroscopy.^15^ The ν_CH_ band is observed at 3152 cm^–1^ at 93 K for the low-spin compound, whereas the same absorption can be found at 3147 cm^–1^ at 298 K for the high-spin compound (see Figure 8). The more subtle changes of the absorption features of the octahedral iron–nitrogen coordination sphere are observed in the FIR spectra (for a temperature-dependent representation of the FIR spectra see Figure S2 in the Supporting Information).

**Figure 9:**
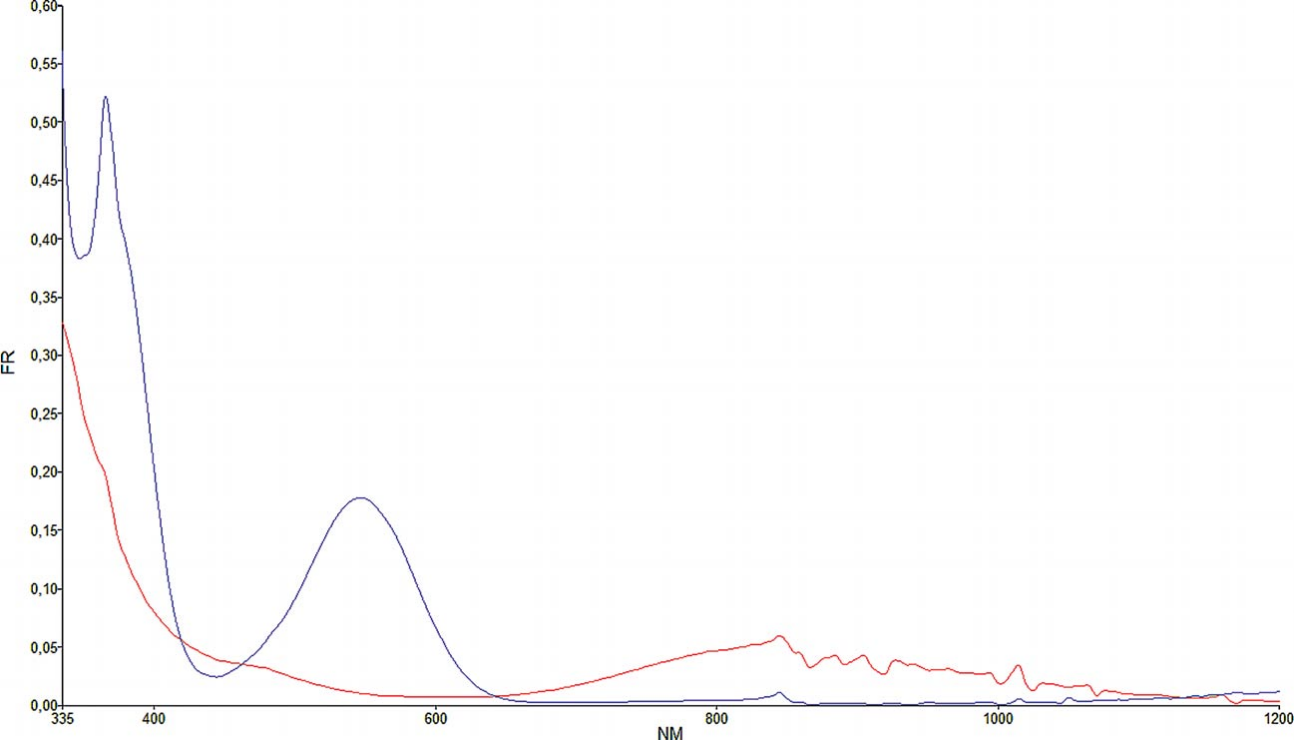
UV/Vis/NIR spectra: a comparison of [Fe(*3ditz*)_3_](BF_4_)_2_ at 138 (black) and 173 K (red), showing the typical d–d transitions for Fe^II^ in the low-spin and the high-spin state, respectively.

The two distinct iron(II) spin states can be easily identified because of the different colors of the HS compound (colorless) and the LS compound (purple), which correspond to different electronic d–d transitions. Therefore, variable-temperature UV/Vis/NIR spectroscopy of the solid powder sample with the diffuse-reflection technique allows for a detection of the electronic absorptions. The HS compound shows no significant absorption in the visible range 400–700 nm, as the absorption corresponding to the ^5^T_2_→^5^E transition, which is characteristic of Fe^II^ HS compounds, arises around 850 nm. In contrast, the purple color of the LS species is due to the ^1^A_1_→^1^T_1_-transition band at 545 nm, which is distinctive for LS Fe^II^ compounds. Another typical absorption feature at 383 nm corresponds to the ^1^A_1_→^1^T_2_ transition and can be found as a shoulder in the spectrum of the LS species. The comparison of the two spectra is shown in Figure 9.

A temperature-dependent representation of the UV/Vis/NIR spectra is given in Figure S3 in the Supporting Information.

**Figure 10:**
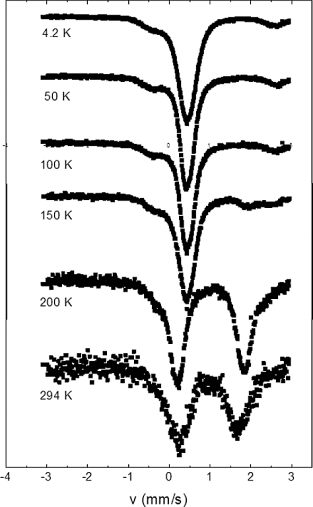
^57^Fe-Mössbauer spectra: a comparison of [Fe(*3ditz*)_3_](BF_4_)_2_ at 4.2, 50, 100, 150, 200, and 294 K shows the transitions of Fe^II^ from the low-spin to the high-spin state.

^57^Fe-Mössbauer spectroscopy is a direct method to determine the spin state of the Fe^II^ coordination centers. For an analysis of the Mössbauer spectra (see Figure 10), four subspectra are necessary.

At high temperature, the whole sample is in the high-spin state. 87 % of the Fe atoms have a quadrupole splitting (QS) *eQV_zz_*/4 = (0.77 ± 0.07) mm/s, the rest has a splitting of (1.1 ± 0.01) mm/s. The center shift (*CS*) for both components is (0.93 ± 0.03) mm/s at 294 K. The transition to the low-spin state appears between 200 and 150 K, which is in good agreement with the results of the magnetic measurements (see Figure 7). At low temperatures, the high-spin component with its higher quadrupole splitting is still present down to the lowest temperatures measured. The rest of the Fe centers changes to the low-spin state with *QS* = 0, splitting into two components. The larger one (70 %) has a center shift *CS* = (0.386 ± 0.02) mm/s; the smaller component exhibits *CS* = (1.102 ± 0.02) mm/s.

## Conclusions

The missing link [Fe(*3ditz*)_3_](BF_4_)_2_ between the complex [Fe(*2ditz*)_3_](BF_4_)_2_ and the complexes [Fe(*nditz*)_3_](BF_4_)_2_ with *n* = 4–9 was only obtained after the strategy of the ligand synthesis was changed. The well-established synthetic pathway of Franke et al.^6^ failed to produce the desired ligand *3ditz* because of unclear problems. The modified approach that makes use of a phase-transfer catalyst could fill the gap in the series of accessible ligands, and thus allowed for a successful preparation and characterization of the new spin-crossover coordination polymer [Fe(*3ditz*)_3_](BF_4_)_2_. Unlike the isostructural 1D chain-type coordination polymers [Fe(*2ditz*)_3_](BF_4_)_2_7a and [Fe(*btzp*)_3_](BF_4_)_2_7b, the title compound features an almost complete and abrupt spin-transition behavior at *T*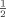 = 159 K, whereas the former complexes showed gradual spin transitions around *T*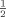 = 140 K and *T*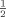 = 130 K, respectively. In most cases, an abrupt spin transition can be observed because of a rigid ligand systems used. Only in a few cases, per se flexible ligands like *3ditz* are packed tightly or establish a rigid coordination-polymer network. In such rare cases, the abruptness of the spin-transition behavior is only due to the strong cooperativity enforced by the whole molecular packing. The title compound is a new example of exactly such a rather rare case.

## Experimental Section

**Warning:** Tetrazoles and their derivatives are potentially shock-sensitive or explosive compoundsand should therefore be handled with great care.

**Spectroscopic Characterization of *3ditz*:** ^1^H NMR and ^13^C NMR spectra in [D_6_]Me_2_CO were measured by using a Bruker 200 FS FT-NMR spectrometer. ^1^H NMR chemical shifts are reported in *ppm* against TMS. Mid-range IR spectra of the ligand were recorded with the attenuated-total-reflectance (ATR) technique within the range 4000–450 cm^–1^ by using a Perkin–Elmer Spectrum Two FTIR spectrometer with a UATR accessory attached.

**Synthesis of 1,3-Bis(tetrazol-1-yl)propane (*3ditz*):** 1,3-Dibromopropane (3.54 mL, 34.67 mmol), sodium hydroxide (2.77 g, 69.35 mmol), and 1*H*-tetrazole (4.86 g, 69.35 mmol) were heated to refluxed in a mixture of 30 mL of toluene and 30 mL of water for 20 h with tetrabutylammoniumbromide (10 mol-%) as phase-transfer catalyst. The solvents were removed in vacuo and the yellow crude oil was purified by column chromatography on silica gel with ethyl acetate as the mobile phase. After evaporation of the solvent, the ligand was obtained as a white solid. Single crystals suitable for X-ray diffraction analysis were grown by slow evaporation from methanol, yield 0.66 g (10.6 %). C_5_H_8_N_8_ (180.17): calcd. C 33.33, H 4.48, N 62.19; found C 33.84, H 4.42, N 61.40. ^1^H NMR (200 MHz, [D_6_]Me_2_CO, 298 K): *δ* = 2.70 (m, *J* = 6.95 Hz, CH_2_), 4.71 (t, *J* = 6.95 Hz, CH_2_), 9.19 (s, CH) ppm. ^13^C NMR (50 MHz, [D_6_]Me_2_CO, 298 K): *δ* = 30.47 (CH_2_), 45.77 (CH_2_), 144.46 (CH) ppm. IR: 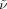 = 3144, 3122, 1568, 1486, 1428, 1171, 1112, 1102, 874, 664, 642 cm^–1^.

**Characterization of [Fe(*3ditz*)_3_](BF_4_)_2_:** Elemental analyses (C, H, N) were performed at the Mikroanalytisches Laboratorium, University of Vienna, Vienna, Austria. Mid-range IR spectra of the complex were recorded by using KBr pellets within the range 3400–600 cm^–1^ with a Perkin–Elmer 400 FIR/MIR FTIR spectrometer. Pellets were obtained by pressing the powdered mixture of the samples in KBr in vacuo by using a Carver 4350.L hydraulic press and by applying a pressure of approximately 10.000 kg/cm^2^ for 5 min. Far-range IR spectra were recorded within the range 700–30 cm^–1^ with the same Perkin–Elmer 400 FIR/MIR FTIR spectrometer and by using polyethylene pellets. The variable-temperature IR spectra were recorded by using a Graseby Specac thermostatable sample holder. Electronic spectra of the undiluted powder samples were measured with a Perkin–Elmer Lambda 900 UV/Vis/NIR spectrometer equipped with a thermostatable powder sample holder in diffuse reflection geometry (Praying Mantis accessory®) between 335 and 1200 nm within the temperature range 138–173 K. Magnetic measurements were performed by using a cryogenic vibrating-sample magnetometer (VSM) between 50 and 350 K. For TGA/DSC (TGA: thermogravimetric analysis, DSC: differential scanning calorimetry) analyses a Netzsch STA 449 F1 Jupiter system with a heating rate of 10 K/min under N_2_ atmosphere was used between 298 and 673 K. ^57^Fe-Mössbauer measurements were performed with a standard constant-acceleration spectrometer in transmission geometry. The ^57^Co/Rh source was mounted on the driving system and kept at room temperature. All center shift data are given relative to this source. The calibration of the velocity scale was carried out with a α-Fe foil. For the temperature variation between 4.2 K and room temperature, a continuous-flow cryostat was used, in which the sample is kept in He exchange gas. The temperature stability was ±0.5 K at temperatures above 77 K and ±0.2 K below. The spectra were analyzed by solving the full Hamiltonian with both magnetic and quadrupole hyperfine interaction.

**Synthesis of the [Fe(*3ditz*)_3_](BF_4_)_2_:** For all manipulations of iron(II) salts, standard Schlenk techniques were used. Fe(BF_4_)_2_**·**6H_2_O (236 mg, 0.6 mmol) and *3ditz* (324 mg, 1.8 mmol) were heated in 10 mL of methanol for 6 h at 45 °C. After the evaporation of the solvent, the white residue was washed twice with 3 mL of dichloromethane. Single crystals suitable for X-ray diffraction analysis were grown by slow evaporation of a crude reaction mixture, yield 163 mg (35.4 %). C_15_H_24_B_2_F_8_FeN_24_ (769.97): calcd. C 23.40, H 3.14, N 43.66; found C 22.57, H 3.11, N 40.75.

**Simultaneous Thermal Analysis:** Explosive decomposition at 525 K (727.6 J/g), residual mass (673 K) 33.3 %. For detailed graphical representations of the DSC and TGA experiments see Figures S4 and S5 in the Supporting Information.

***Note:*** The perchlorate analogue was also prepared and showed thermal spin-crossover behavior upon cooling in liquid nitrogen. Because of the very explosive character of this compound, further investigations were not considered.

**Single-Crystal X-ray Diffraction:** Single crystals of the title compounds were attached to a glass fiber by using perfluorinated oil and were mounted on a Bruker KAPPA APEX II diffractometer equipped with a CCD detector. Data were collected at 100 K (both compounds) and 200 K {[Fe(*3ditz*)_3_](BF_4_)_2_} in a dry stream of nitrogen with Mo-*K*_α_ radiation (*λ* = 0.71073 Å). Redundant data sets up to 2*θ* = 65° (*3ditz*) and 55° {[Fe(*3ditz*)_3_](BF_4_)_2_} were collected. Data were reduced to intensity values by using SAINT-Plus^16^, and an absorption correction was applied by using the multiscan method implemented by SADABS.^16^ The structures at 100 K were solved by using charge-flipping implemented by SUPERFLIP.^17^ An initial model of [Fe(*3ditz*)_3_](BF_4_)_2_ at 200 K was derived from a model based on 100 K data. The structures were refined against *F* values with JANA2006.^18^ The assignment of the C atom in the tetrazole rings was unambiguous because of electron density in the difference Fourier maps, which corresponded to the appertaining H atom and because of a distinct worsening of the reliability factors upon wrong assignment. The positions of the protons in *3ditz* were freely refined. For the Fe^2+^ complex, protons were placed at calculated positions and refined as riding on the parent C atoms. All non-H atoms were refined with anisotropic displacement parameters. Important crystallographic data are compiled in Table 2.

CCDC-900354 (for *3ditz*), -CCDC-900355, and -CCDC-912625 {for [Fe(*3ditz*)_3_](BF_4_)_2_ at 100 and 200 K, respectively} contain the supplementary crystallographic data for this paper. These data can be obtained free of charge from The Cambridge Crystallographic Data Centre via www.ccdc.cam.ac.uk/data_request/cif.

**X-ray Powder Diffraction:** As all spectroscopic and magnetic investigations have been performed on powder samples of the title compound, we verified that the powder samples of the complex are identical to the single crystals used for the structure determination. The X-ray powder diffraction measurements were carried out with a Panalytical X'Pert diffractometer in Bragg–Brentano geometry by using Cu-*K*_α__1,2_ radiation, a X′Celerator linear detector with a Ni filter, sample spinning with backloading sample holders, and 2*θ* = 5–70° at *T* = 297 K. By using the structural data of the single crystals of the complex, Rietveld refinements were carried out with the program TOPAS by using the fundamental parameter approach.^19^ Apart from unit cell dimensions, instrumental parameters, and a background polynomial coefficient, a texture parameter was refined. The comparison of the measured XRPD with the calculated XRPD on the basis of the single crystal XRD data is given in Figure S1 in the Supporting Information.

**Supporting Information** (see footnote on the first page of this article): XRPD pattern of [Fe(3ditz)_3_](BF_4_)_2_ in comparison to a calculated XRPD pattern; variable-temperature FIR spectra of [Fe(3ditz)_3_](BF_4_)_2_; variable-temperature UV/Vis/NIR spectra of [Fe(3ditz)_3_](BF_4_)_2_; DSC during thermal decomposition of [Fe(3ditz)_3_](BF_4_)_2_; TGA during thermal decomposition of [Fe(3ditz)_3_](BF_4_)_2_.

## Supplementary material

As a service to our authors and readers, this journal provides supporting information supplied by the authors. Such materials are peer reviewed and may be re-organized for online delivery, but are not copy-edited or typeset. Technical support issues arising from supporting information (other than missing files) should be addressed to the authors.

## References

[1a] Kahn O, Kröber J, Jay C (1992). Adv. Mater.

[1b] Hauser A, Gütlich P, McCleverty JA, Meyer TJ (2004). Comprehensive Coordination Chemistry II.

[2a] Gütlich P, Goodwin HA (2004). Spin-Crossover in Transition Metal Compounds I–III.

[2b] Gütlich P, van Koningsbruggen PJ, Renz F (2004). Struct. Bonding (Berlin).

[2c] Gütlich P, Goodwin HA, McCleverty JA, Meyer TJ (2004). Comprehensive Coordination Chemistry II.

[3] Dirtu MM, Rotaru A, Gillard D, Linares J, Codjovi E, Tinant B, Garcia Y (2009). Inorg. Chem.

[4] Gandolfi C, Morgan GG, Albrecht M (2012). Dalton Trans.

[5a] Stassen AF, Grunert M, Dova E, Müller M, Weinberger P, Wiesinger G, Schenk H, Linert W, Haasnoot JG, Reedijk J (2003). Eur. J. Inorg. Chem.

[5b] Grunert CM, Weinberger P, Schweifer J, Hampel C, Stassen AF, Mereiter K, Linert W (2005). J. Mol. Struct.

[5c] Absmeier A, Bartel M, Carbonera C, Jameson GNL, Weinberger P, Caneschi A, Mereiter K, Letard J-F, Linert W (2006). Chem. Eur. J.

[5d] Valtiner M, Paulsen H, Weinberger P, Linert W (2007). MATCH Commun. Mathematical Computer Chem.

[5e] Hassan N, Weinberger P, Werner F, Mereiter K, Molnar G, Bousseksou A, Valtiner M, Linert W (2008). Inorg. Chim. Acta.

[5f] Hassan N, Weinberger P, Kubel F, Molnar G, Bousseksou A, Dlhan L, Boča R, Linert W (2009). Inorg. Chim. Acta.

[6] Franke PL, Haasnoot JG, Zuur AP (1982). Inorg. Chim. Acta.

[7a] Schweifer J, Weinberger P, Mereiter K, Boca M, Reichl C, Wiesinger G, Hilscher G, van Koningsbruggen PJ, Kooijman H, Grunert M, Linert W (2002). Inorg. Chim. Acta.

[7b] van Koningsbruggen PJ, Garcia Y, Kahn O, Fournes L, Kooijman H, Spek AL, Haasnoot JG, Moscovici J, Provost K, Michalowicz A, Renz F, Gütlich P (2000). Inorg. Chem.

[7c] Grunert CM, Schweifer J, Weinberger P, Linert W, Mereiter K, Hilscher G, Muller M, Wiesinger G, van Koningsbruggen PJ (2004). Inorg. Chem.

[8a] van Koningsbruggen PJ, Garcia Y, Kooijman H, Spek AL, Haasnoot JG, Kahn O, Linares J, Codjovi E, Varret F (2001). J. Chem. Soc., Dalton Trans.

[8b] Jameson GNL, Werner F, Bartel M, Absmeier A, Reissner M, Kitchen JA, Brooker S, Caneschi A, Carbonera C, Létard J-F, Linert W (2009). Eur. J. Inorg. Chem.

[8c] Bartel M, Absmeier A, Jameson GNL, Werner F, Kato K, Takata M, Boca R, Hasegawa M, Mereiter K, Caneschi A, Linert W (2007). Inorg. Chem.

[9] Gao Z, Zhu X (2012). Huaxue Shiji.

[b20] Gao Z, Zhu X (2012). Huaxue Shiji.

[10] Zatsepina MV, Artamonova TV, Koldobskii GI (2008). Russ. J. Org. Chem.

[11] Katritzky AR, Cai C, Meher NK (2007). Synthesis.

[12a] Quesada M, Prins F, Roubeau O, Gamez P, Teat SJ, van Koningsbruggen PJ, Haasnoot JG, Reedijk J (2007). Inorg. Chim. Acta.

[12b] Bialonska A, Bronisz R, Weselski M (2008). Inorg. Chem.

[12c] Bialonska A, Bronisz R (2008). Tetrahedron.

[13] Kálmán A, Párkányi L (1993). Acta Crystallogr., Sect. B.

[14] van Koningsbruggen PJ, Grunert M, Weinberger P (2003). Monatsh. Chem.

[15] Weinberger P, Grunert M (2004). Vib. Spectrosc.

[17] Palatinus L, Chapuis G (2007). J. Appl. Crystallogr.

[18] Petricek V, Dusek M, Palatinus L

