# Supplementary material for: A Modified Synthetic Pathway for the Synthesis of so far Inaccessible N1-Functionalized Tetrazole Ligands – Synthesis and Characterization of the 1D Chain-Type Spin Crossover Compound [Fe(3ditz)3](BF4)2
Source: Eur J Inorg Chem. 2013 Jan 17;2013(5-6):984–91. doi: 10.1002/ejic.201201062 (PMC3593040; doi:10.1002/ejic.201201062)
Supplement: Supplementary file 1 [file ejic2013-0984-SD1.pdf]

Eur. J. Inorg. Chem. **2013** · © WILEY-VCH Verlag GmbH & Co. KGaA, 69451 Weinheim, 2013 · ISSN 1434–1948

SUPPORTING INFORMATION

DOI: 10.1002/ejic.201201062
Title: Modified Synthetic Pathway for the Synthesis of so far Inaccessible N1-Functionalized Tetrazole Ligands – Synthesis and Characterization of the 1D Chain-Type Spin Crossover Compound $[Fe(3ditz)_3](BF_4)_2$
Author(s): Danny Müller, Christian Knoll, Berthold Stöger, Werner Artner, Michael Reissner, Peter Weinberger*

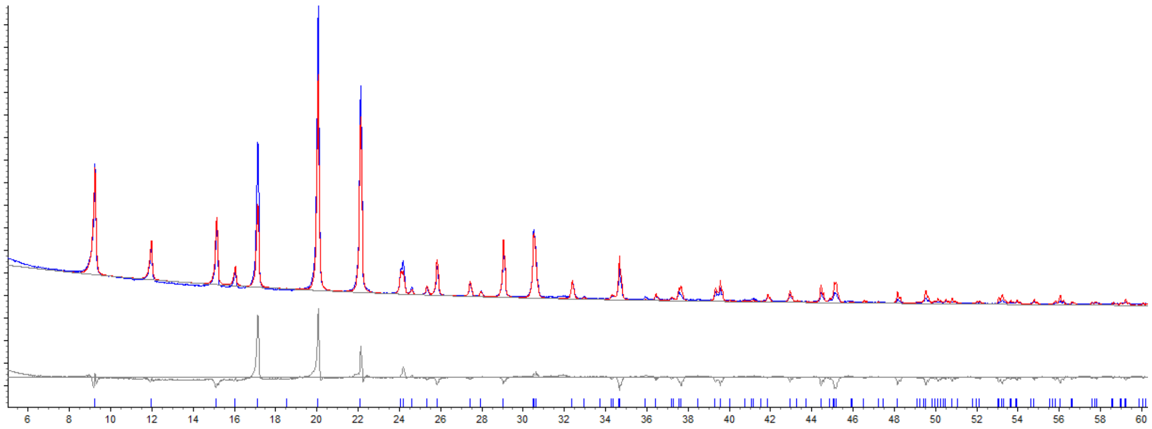

Figure S1: XRPD of [Fe(*3ditz*)$_3$](BF$_4$)$_2$ in comparison to a calculated XRPD pattern using data of the single crystal XRD measurement

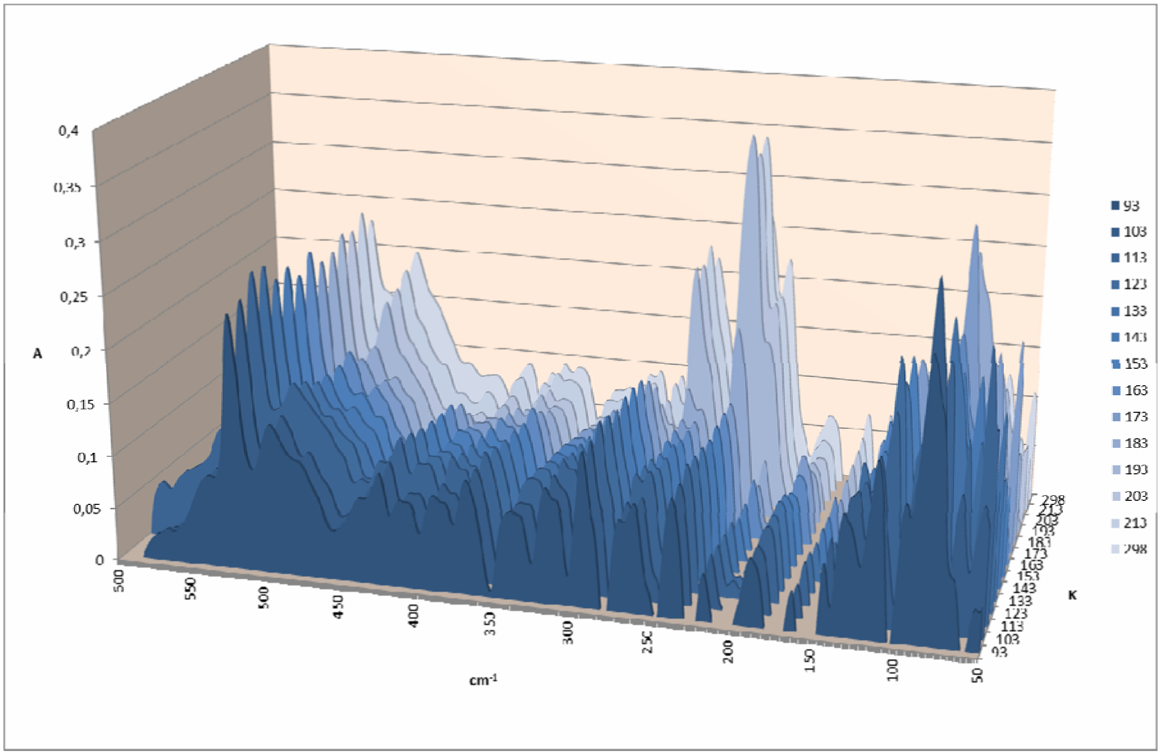

Figure S2: Variable temperature FIR of [Fe(*3ditz*)$_3$](BF$_4$)$_2$

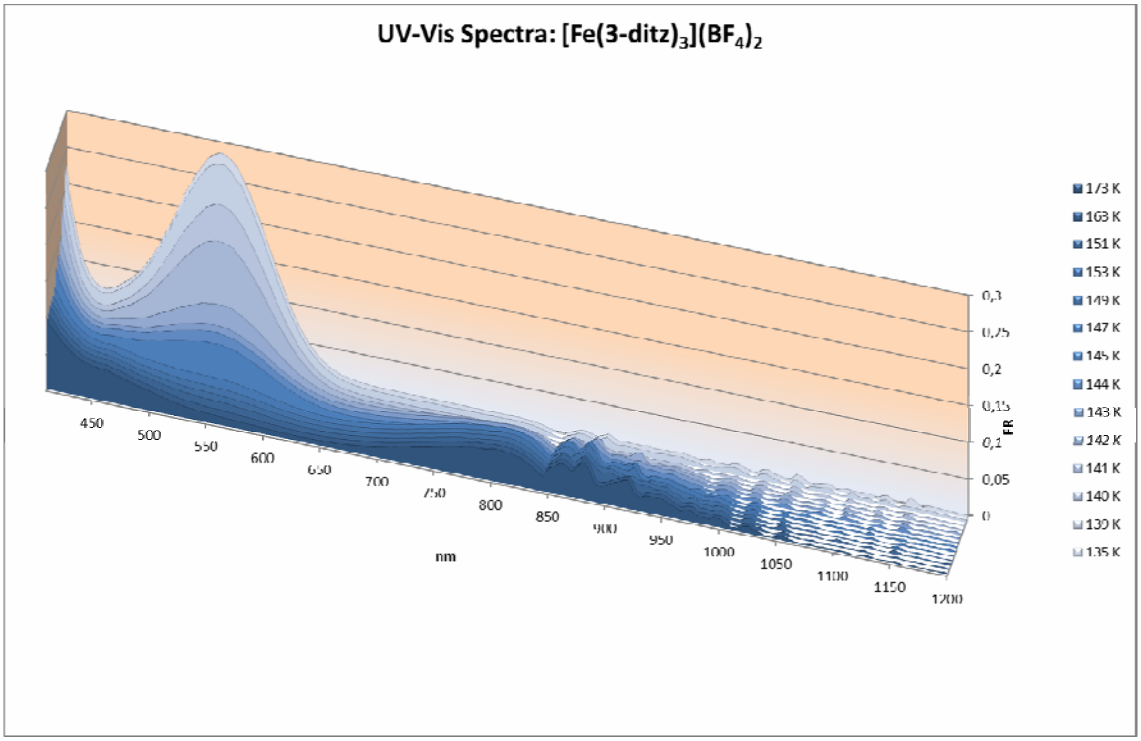

Figure S3: Variable temperature UV-VIS-NIR of $[Fe(\textit{3ditz})_3](BF_4)_2$

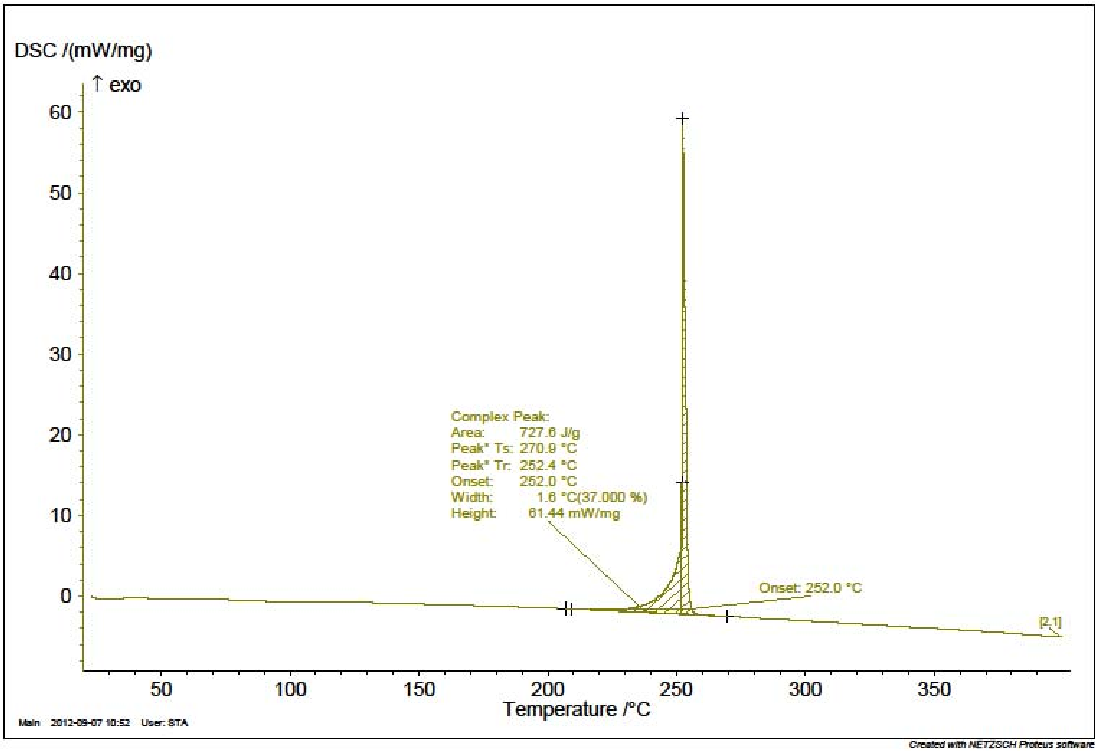

Figure S4: DSC during thermal decomposition of [Fe(*3ditz*)$_3$](BF_4)$_2$

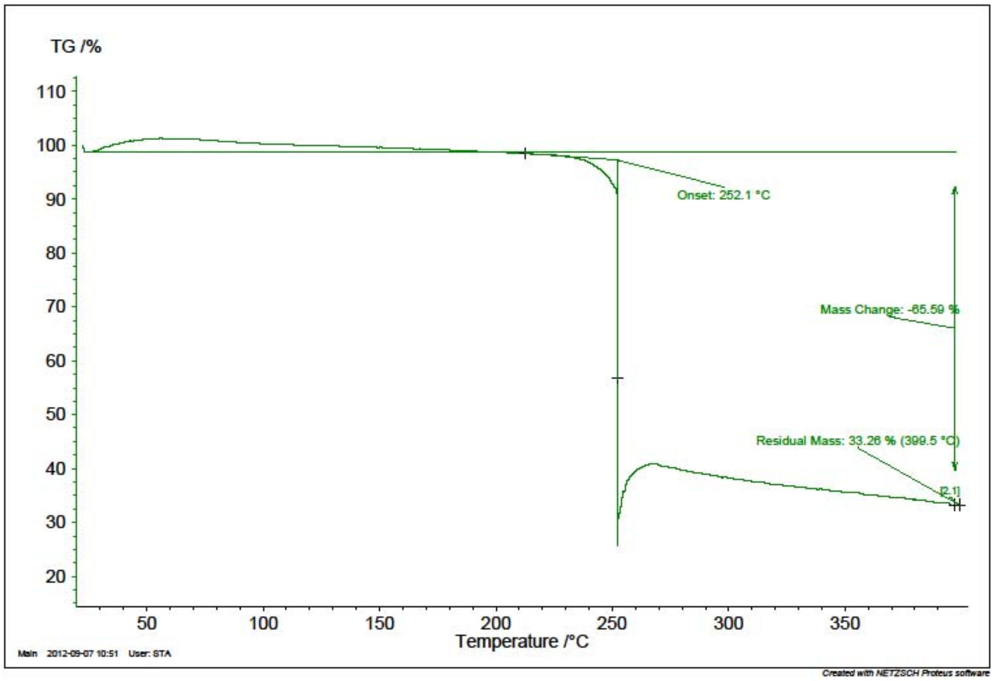

Figure S5: TGA during thermal decomposition of [Fe(*3ditz*)$_3$](BF_4)$_2$
